# Suicide in circumpolar regions: an introduction and overview

**DOI:** 10.3402/ijch.v74.27349

**Published:** 2015-03-04

**Authors:** T. Kue Young, Boris Revich, Leena Soininen

**Affiliations:** 1 School of Public Health, University of Alberta, Edmonton, AB, Canada; 2 Laboratory of Environmental Health, Institute for Forecasting, Russian Academy of Science, Moscow, Russian Federation; 3 Department of Public Health, Hjelt Institute, University of Helsinki, Helsinki, Finland

**Keywords:** Arctic, suicide, mortality, youth

## Abstract

This extended editorial introduces the Special Issue on Suicide and Resilience in Circumpolar Regions, the results of the knowledge synthesis project by an international research team funded by the Canadian Institutes of Health Research and endorsed by the Arctic Council. It focuses on the extent and magnitude of the problem of suicidal behaviours and thoughts from a circumpolar perspective – the variation across Arctic States and their northern regions, the excess risk among some indigenous groups and their demographic characteristics. Much remains to be learned about the design and implementation of youth-focused intervention programmes, especially in a circumpolar comparative framework.

Suicide among indigenous youth has emerged as a serious public health challenge in circumpolar regions over the past several decades. To date, the problem has shown no sign of abating, although much research has been done to understand its causes and various interventions have been attempted in many localities (1–3). A significant step forward was the Hope and Resilience: Suicide Prevention in the Arctic Conference that was convened in Nuuk, Greenland, in November 2009 (4). The conference was an example of mutual learning and sharing of best practices among circumpolar regions. Unique in being youth oriented, it featured panel discussions where youth representatives challenged government ministers and other policy makers.

In February 2011, Health Ministers and representatives from Member States of the Arctic Council signed the Arctic Health Declaration in Nuuk. It specifically pledged to “enhance mental health and prevention of substance abuse and suicide through exchange of experiences and good practices” (5). In 2014, the Canadian Institutes of Health Research launched a funding programme to support the creation of international teams to synthesize current knowledge, develop the evidence base and identify promising practices for scaling up by Arctic regional governments and indigenous organizations.

This special issue of the *International Journal of Circumpolar Health* presents results from the research by one of the international teams led by Susan Chatwood of the Institute for Circumpolar Health Research based in Yellowknife, Northwest Territories, Canada. The collection of papers include epidemiological analyses, a scoping review of existing interventions and a multiple case study of projects, programmes and policies in northern Canada, Greenland and northern Norway.

Over the years *IJCH* has published articles on suicide prevention, including case studies and systematic reviews, as well as a special issue devoted to this topic in 2009.

This introductory article to the current Special Issue sets the scene by describing the magnitude and extent of the problem from a circumpolar perspective, with special focus on the indigenous populations.

First a methodological note: When comparing suicide rates (i.e. mortality rates due to suicide), the statistical procedure of age standardization is used. It ensures that the rates are comparable across populations with widely different age structure and where certain age groups are more prone to the health problem than others. Different “standard populations” can be used. When comparing all circumpolar regions (as in Figs. 1 and 2), the hypothetical European Standard Population is used. However, for other datasets, especially those where only published rates are reported and no details are available to recalculate age-standardized rates, a different standard population may be used, for example, the 2000 US population (as in Fig. 3). Sometimes the crude rates are reported, as the original author did not use age standardization. Readers should be cautioned that rates cannot be compared across tables and figures which use different standard populations. It should also be recognized that some circumpolar regions have relatively small populations and that suicides are rare events, resulting in fluctuations in rates from year to year. By aggregating data over a 10-year period, the instability of rates is reduced.

**Fig. 1:**
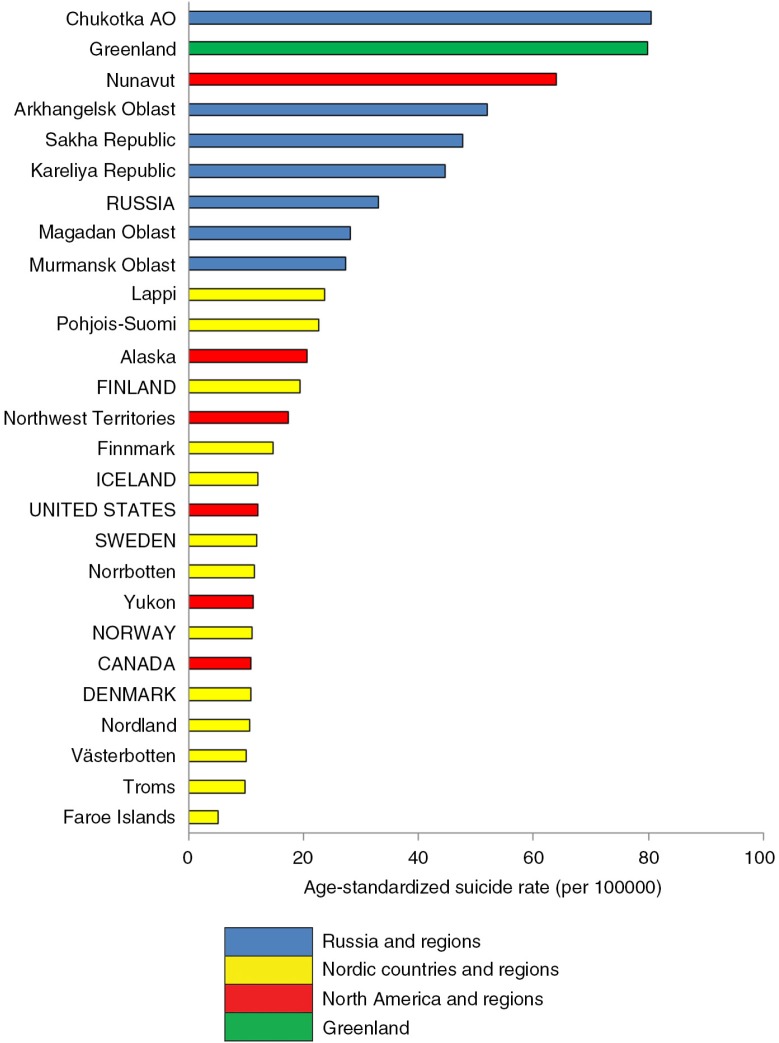
Mean age-standardized suicide rate in the 8 Arctic States and their northern regions for the decade 2000–2009. Note: Direct age-standardization to the European Standard Population. Sources: National statistical agencies including the National Center of Health Statistics (USA), Statistics Canada, Statistics Iceland, Statistics Norway, Socialstyrelsen (Sweden), Statistics Finland and Rosstat (Russia); and international databases (NOMESCO, Eurostat).

**Fig. 2:**
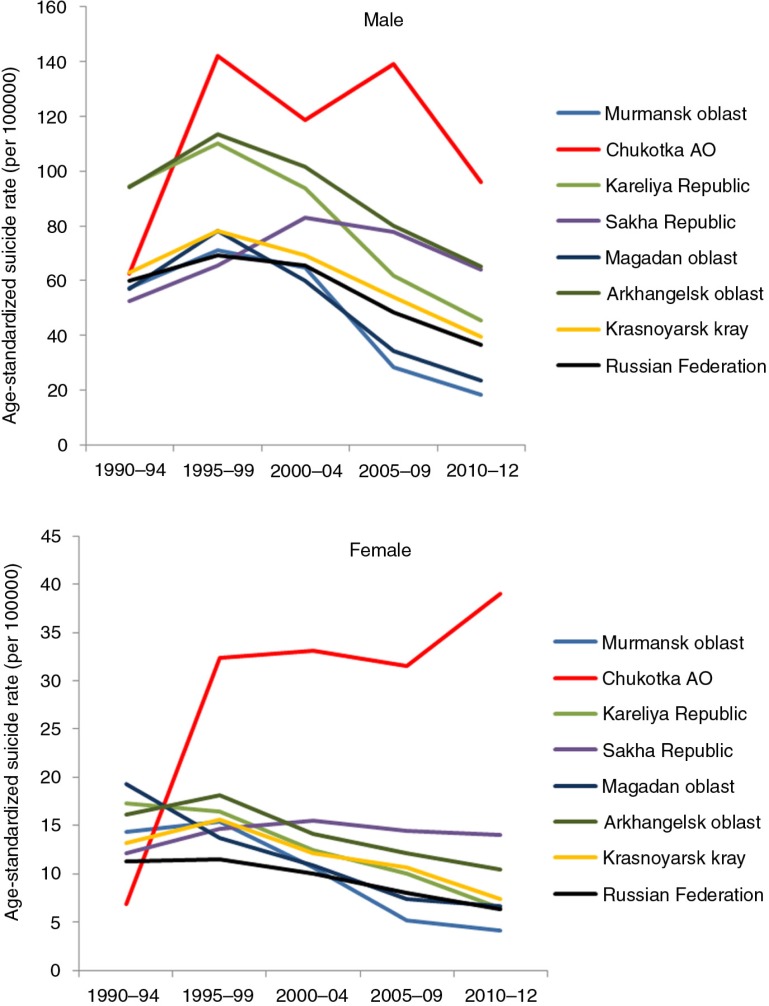
Time trend in suicide rates in Russia and selected northern regions, 1990–2012. Source: unpublished data from Rosstat. Note: Suicide rate directly age standardized to the European Standard Population.

**Fig. 3:**
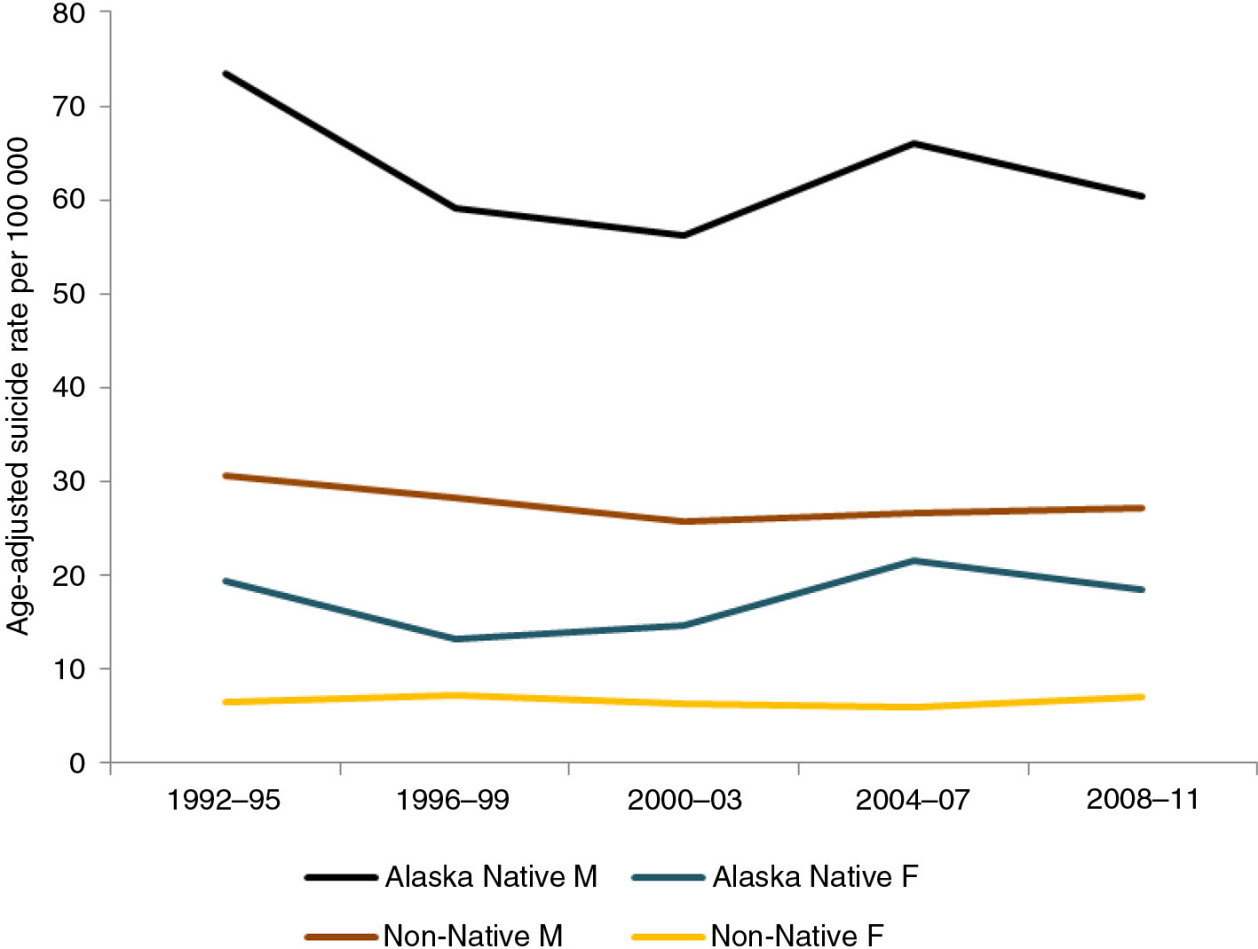
Time trend in sex-specific suicide rate in Alaska: Native versus non-Native. Source: Alaska Native Tribal Health Consortium (6). Note: Age-standardized to the US 2000 population.

## Regional variation

Figure 1 presents the mean age-standardized suicide rate for the decade 2000–2009 in the 8 Arctic States (members of the Arctic Council – Canada, Denmark, Finland, Iceland, Norway, Sweden, Russian Federation and United States of America) and their northern regions (for which data are available). Note that the national and regional populations are inclusive of all ethnicities. The top three ranked regions are Chukotka, Greenland and Nunavut. These are also regions with a high proportion of the population who are indigenous – approximately 90% in Greenland, 85% in Nunavut, and 30% in Chukotka. The Nordic countries and their northern regions occupy the low end of the spectrum, with the exception of Finland and its 2 northern regions, where suicide rates are higher than other Nordic countries and regions.

Suicide in Russia and its regions rose and peaked in the latter half of the 1990s, which appears to correspond to the substantial social and economic dislocations of the early post-Soviet era. However, the rates in Chukotka outstripped that of other regions, especially among women (Fig. 2).

## Indigenous populations

Among the circumpolar Inuit, the increase in suicide rates occurred first among Alaska Natives, later in Greenland, and still later in the Nunavik and Baffin regions in the eastern Canadian Arctic (4), with each later “epidemic” more severe than the preceding one. A more in-depth review of the situation in Greenland is provided by Bjerregaard and colleagues in a paper in this Special Issue.

In a jurisdiction where mortality data can be segregated by ethnicity/race, as in Alaska, the excess risk of suicide in the indigenous population is clearly evident (Fig. 3). Between 2002 and 2011 the Alaska Native rate was on average 2.4 times the non-Native in the state. There was also substantial regional variation within Alaska, with the highest rate in the Norton Sound region (inhabited by Inupiat) as much as 4 times the lowest rate in the Southeast inhabited by Northwest Coast Indians (6).

**Fig. 4:**
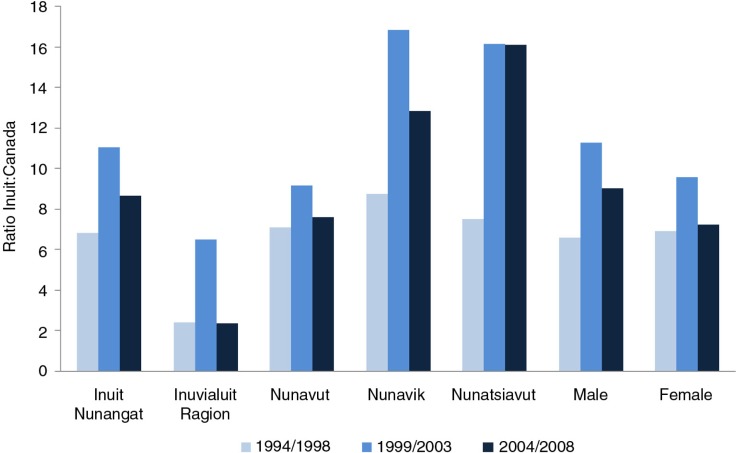
Risk of suicide among Canadian Inuit relative to all Canadians. Source: Statistics Canada. CANSIM Table 102-0704 (7). Note: Ratio refers to the ratio of age-standardized suicide rate in an Inuit region to the national rate in Canada. Inuit Nunangat refers to the traditional Inuit homeland in Canada and is composed of the Inuvialuit Region in the Northwest Territories, Nunavut Territory, the Nunavik region in northern Québec province and the Nunatsiavut region in the province of Newfoundland and Labrador.

Among Canadian Inuit, the excessive risk of suicide is present in all 4 Inuit regions, and in both males and females (7). Over the three 5-year periods, the peak occurred during the middle period 1999–2003 and appeared to have declined in the most recent period (Fig. 4). Note that the cases and populations strictly do not refer to ethnic Inuit, but to those residents of communities defined as predominantly Inuit based on the “geozone method” using geographical and demographic criteria (8).

**Fig. 5:**
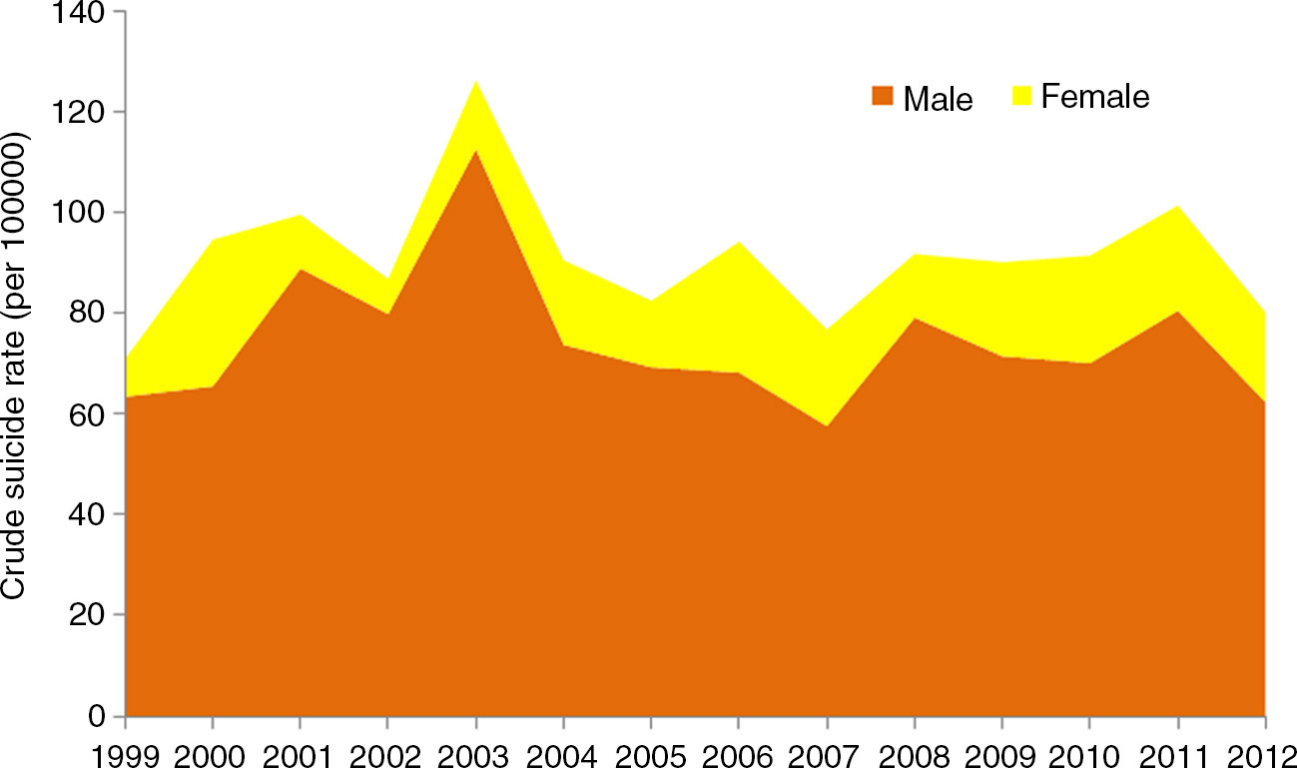
Distribution of suicide cases by sex in Nunavut, 1999–2012. Note: The relative size of the 2 coloured areas represent the contribution of male and female cases to the overall crude suicide rate for Nunavut, that is, the line on top of the coloured areas. Source: Nunavut Bureau of Statistics (16).

The only study on an indigenous group in Arctic Russia – the Nenets in the Nenets Autonomous Okrug in northwestern Russia – also showed a higher mortality than non-Nenets in the region (9). In Yakutia, ethnic Yakuts accounted for 45.5% of the population but 62.2% of suicide cases between 2011 and 2013. Slavic people accounted for 42% of the population and 32% of the cases. Ethnic-specific rates, however, are not available (10).

Ethnic identity is not recorded in health and social databases in the Nordic countries. Information on suicide among the Sami is derived from studies where Sami identity among study participants was specifically determined based on a variety of linguistic, genealogical and socio-political criteria. Regional Sami cohorts have been assembled in Norway (11) covering the period 1970–1998, Sweden (12) covering the period 1961–2000 and Finland (13) covering the period 1979–2005, which was updated to 2010 by one of us (LS). Table I summarizes the standardized mortality ratios for suicide, comparing Sami with non-Sami in the same regions or nationally, in the 3 cohorts. A ratio greater than 1.0, and if its 95% confidence interval does not enclose 1.0, indicates that the Sami rate is higher than the non-Sami, having adjusted for the different age structures of the 2 populations.

**Table I:** Risk of suicide among Sami relative to non-Sami in northern Norway, Sweden and Finland

	Cohort	M	F
Northern Sweden	Total cohort	1.17^a^	0.76^a^
	Non-herding	1.05	0.67
	Herding	1.50	1.12
Northern Norway	Total cohort	1.27	1.27
	Finnmark	1.50^a^	1.55
	Troms	0.74	1.00
	Nordland	0.42	3.17
	Core area	1.54^a^	1.31
	Coast	1.24	1.21
	South	0.41	1.51
	1970–1980	1.17	1.14
	1981–1990	1.36	1.92
	1991–1998	1.20	0.81
	Non-herding	1.30^a^	1.34
	Herding	1.06	0.66
Northern Finland	Total cohort	1.78^a^	1.26
	1979–1987	1.83	No case
	1988–1996	1.07	1.93
	1997–2005	2.55^a^	1.2
	2006–2010	2.32	1.2

Swedish data from Hassler et al. (12), Norwegian data from Silviken et al. (11) and Finnish data from Soininen and Pukkala (13) updated to 2010 for this article. Ratios in table refer to Standardized Mortality Ratios;

a refers to ratios with 95% confidence intervals not including unity.

Unlike other health indicators, where disparities between Sami and non-Sami are very small or non-existent (14), there is an excess of suicide among Sami. Among men, the excess ranged from only 17% higher in the Swedish cohort to as much as 2.5 times higher in the Finnish cohort during the period 1997–2005. On the other hand, there is no excess suicide risk among Sami women in any of the 3 cohorts.

Firearms and hanging tend to be the method of choice for suicide among indigenous people in the Arctic (11,15) . The use of firearms can be attributed to the widespread ownership of firearms for hunting, both as a traditional pursuit and form of modern recreation. Hanging tends to be favoured more by women.

## Age–sex distribution

Suicide is predominantly a phenomenon among men in the Arctic as elsewhere. Figure 5 shows the distribution by sex in Nunavut (16). A higher male rate is observed in both the Native and non-Native populations of Alaska (Fig. 3) and in Russia and its regions (Fig. 2).

**Fig. 6:**
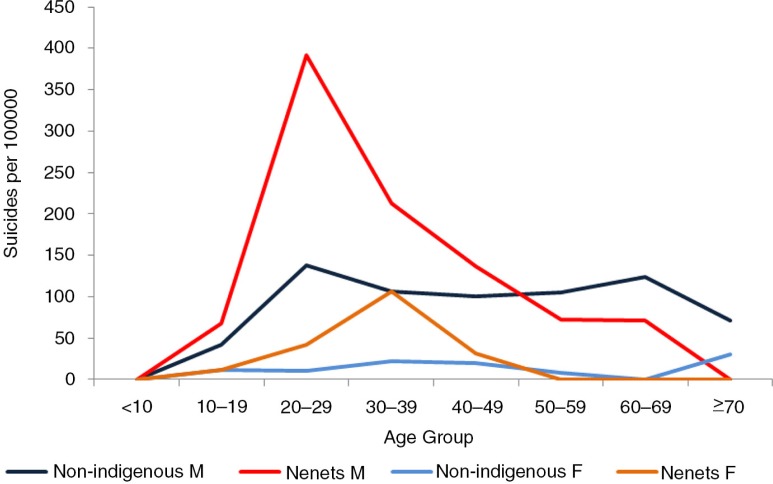
Age–sex distribution of suicide among the Nenets in northwestern Russia. Source: Sumarokov et al. (9).

**Fig. 7:**
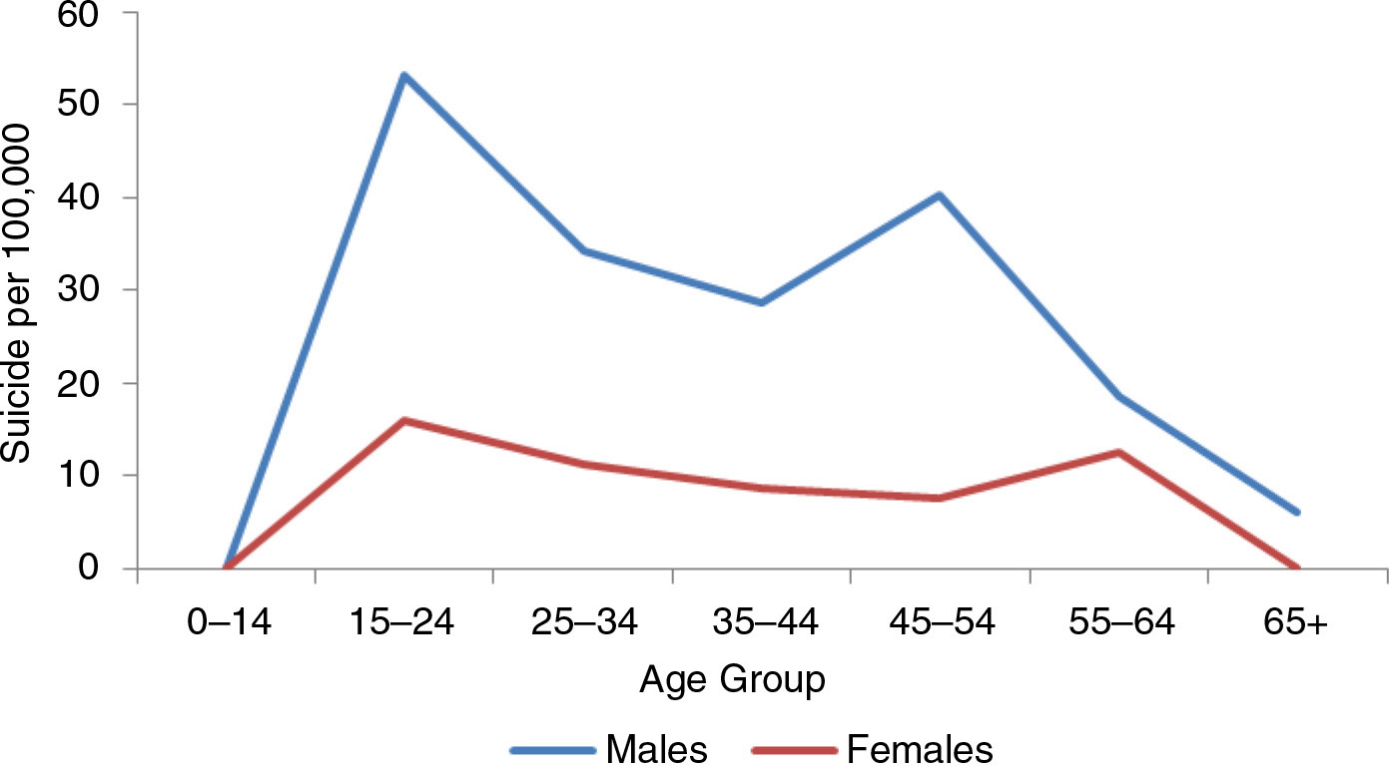
Age–sex distribution of suicide among Norwegian Sami. Source: Data from the northern Norwegian cohort from 1970 to 1998 reported by Silviken et al. (10).

The age–sex pattern of suicide among the Nenets in Russia is similar to that observed in other Arctic populations (9) (Fig. 6). The Sami pattern, however, shows 2 peak ages (11) – the peak at age 15–24 is similar to that seen in other Arctic indigenous populations, whereas the peak at older age group 45–54 is more typical of that of non-indigenous people (Fig. 7).

## Suicidal attempts and thoughts

For every successful suicide, there are many more suicide attempts, and for every suicide attempt there are numerous people harbouring suicidal thoughts (17–19). The suicide attempts may or may not result in any contact with the health and social service systems and their true magnitude is thus difficult to estimate. The distinction is not sharp between overt suicidal behaviour and other self-destructive behaviour such as excessive risk-taking or alcohol and drug abuse in which the likelihood of dying is high. Many accidental deaths may result from a concealed wish to die.

**Fig. 8:**
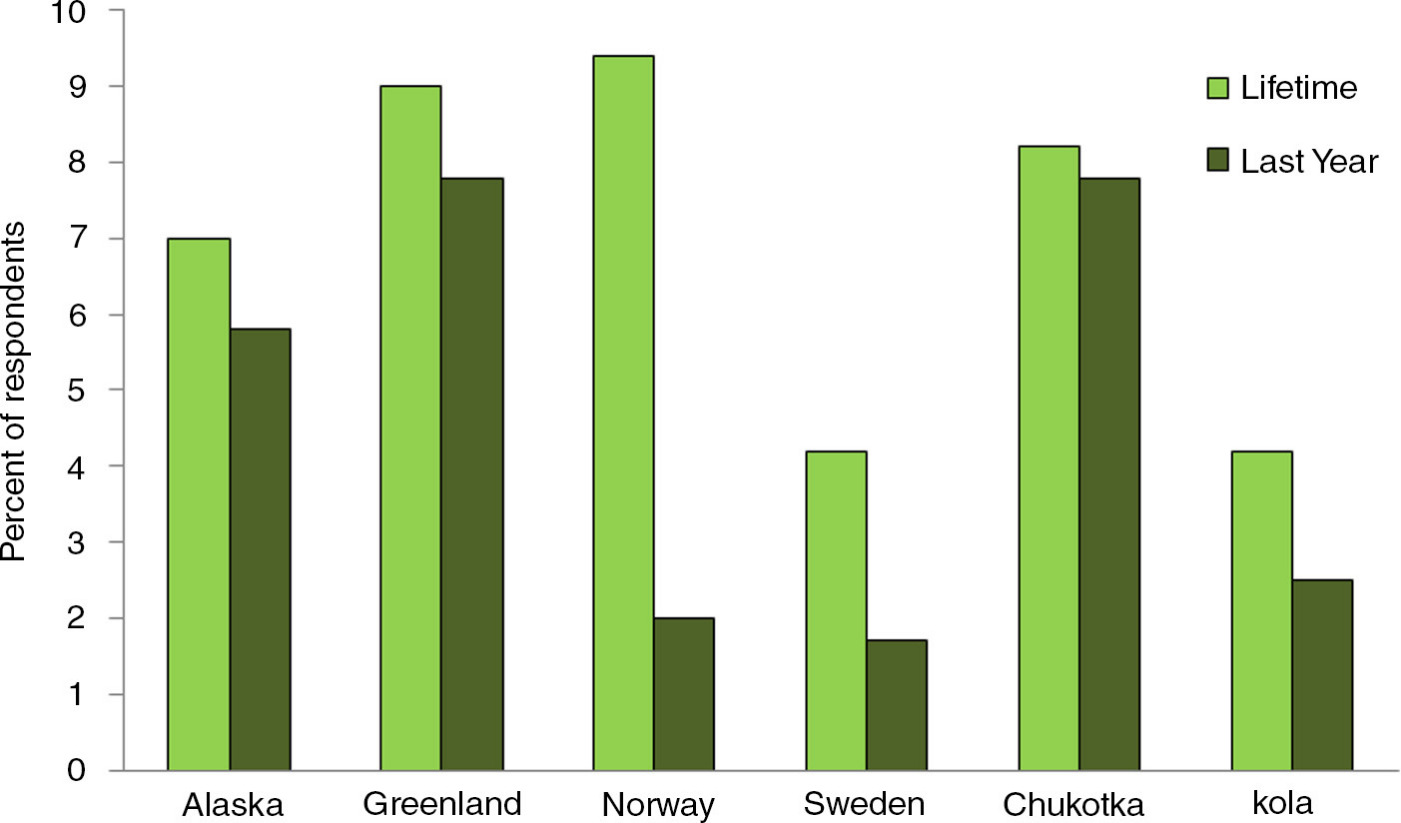
Proportion of respondents who had seriously thought of committing suicide in their lifetime and within the last year, indigenous people in selected regions. Source: Survey of Living Conditions in the Arctic (SLiCA), as presented by B. Poppel in Fig. 3 of *Hope and Resilience Conference Report* (4).

The Survey on Living Conditions in the Arctic (SLiCA) among indigenous people in several regions (20) provides some measure of the extent of suicidal ideation (in the past year and over the lifetime) (Fig. 8).

In Canada, preliminary data from the Inuit Health Survey conducted in 2007–2008 (21) found that 48% of adults in Nunavut had seriously thought of suicide at some point in their lifetime, and 14% within the past year. Those who had actually attempted suicide at some point in their life were 29%, and those who did so within the past year was 5%. Overall, the prevalence was higher in the younger age groups and among women. The corresponding figures for the Dene in the Northwest Territories tend to be lower than among the Inuit in the Nunavut – 14% with lifetime suicidal thoughts (and 5% within the past year), and 8% with lifetime history of suicide attempt (and 1% within the past year) (22).

The female preponderance among those who had suicide thoughts and suicide attempts in Arctic populations is similar to that observed in other populations globally.

## Suicide intervention

Suicide intervention programmes come in different shapes and forms, and may range from individually focused actions such as helping those who attempt suicide, identifying and treating depression, and reducing substance use, to broader legislative actions such as those controlling firearms availability. Programme evaluation to demonstrate any measurable effect in reducing suicidal behaviours or long-term impact has rarely been performed or published, as will be made clear in the scoping review by Chatwood and colleagues in this Special Issue. The Hope and Resilience Conference presented examples of local initiatives in Alaska, Canada, Greenland and Norway (4). Young people tend not to seek help from official health services agencies. Mental health services thus have a special responsibility to make their services more accessible and attractive to young people.

Efforts need to be directed at the entire community, especially in identifying resilience and protective factors, and not only focusing on individuals who are at risk. Political self-determination is often held out as a panacea for health improvement, yet the world's highest youth suicide rates occur in Greenland and Nunavut, 2 jurisdictions where indigenous people have advanced the furthest towards self-government. Such issues are often not on the mind of youths contemplating suicide. Studies and workshops have shown that adults may identify boredom as the main reason for suicide, and the solution would be programmes offering recreational activities, job training and strengthening traditional culture. In contrast, youths may attribute suicide to stress and emphasize the need for meaningful everyday communication and interaction, more attentive parents and less alcohol abuse. There is still much to be learned about the design and implementation of youth-focused intervention programmes. It is hoped that this Special Issue will provide the impetus towards such efforts.

## References

[1] Lehti V, Niemelä S, Hoven C, Mandell D, Sourander A (2009). Mental health, substance use and suicidal behaviour among young indigenous people in the Arctic: a systematic review. Soc Sci Med.

[2] MacDonald JP, Ford JD, Willox AC, Ross NA (2013). A review of protective factors and causal mechanisms that enhance the mental health of Indigenous circumpolar youth. Int J Circumpolar Health.

[3] Allen J, Hopper K, Wexler L, Kral M, Rasmus S, Nystad K (2014). Mapping resilience pathways of indigenous youth in five circumpolar communities. Transcult Psychiatry.

[4] Larsen CVL, Pedersen CP, Berthelsen SW, Chew C (2009). Hope and resilience: suicide prevention in the Arctic. http://www.paarisa.gl/media/18092/seminarreport_hope_and_resilience_final_26_10_10.pdf.

[5] Arctic Council Secretariat (2011). Arctic health declaration, Nuuk, Greenland. http://www.arctic-council.org/index.php/en/document-archive/category/196-5-human-development.

[6] Alaska Native Tribal Health Consortium (2014). Alaska native injury atlas: an update. http://www.anthctoday.org/epicenter/publications/injury_atlas/index.html.

[7] Statistics Canada Mortality, by selected causes of death (ICD-10) and sex, five-year average, Canada and Inuit regions, every 5 years. CANSIM Table 102-0704 [Accessed 2014 Dec 29]. http://www5.statcan.gc.ca/cansim/home-accueil?lang=eng.

[8] Peters PA, Oliver LN, Carrière GM (2012). Geozones: an area-based method for analysis of health outcomes. Health Rep.

[9] Sumarokov YA, Brenn T, Kudryavtsev AV, Nilssen O (2014). Suicides in the indigenous and non-indigenous populations in the Nenets Autonomous Okrug, Northwestern Russia, and associated socio-demographic characteristics. Int J Circumpolar Health.

[10] Yakovlev MV, Kolbina EY (2014). Specific features of suicidal patterns in the Sakha Republic (Yakutia): actual questions of suicidology [In Russian].

[11] Silviken A, Haldorsen T, Kvernmo S (2006). Suicide among indigenous Sami in Arctic Norway, 1970–1998. Psychiatr Epidemiol.

[12] Hassler S, Johansson R, Sjölander P, Grönberg H, Damber L (2005). Causes of death in the Sami population of Sweden, 1961–2000. Int J Epidemiol.

[13] Soininen L, Pukkala E (2008). Mortality of the Sami in northern Finland 1979–2005. Int J Circumpolar Health.

[14] Sjölander P (2011). What is known about the health and living conditions of the indigenous people of northern Scandinavia, the Sami?. Glob Health Action.

[15] Bjerregaard P, Lynge I (2006). Suicide: a challenge in modern Greenland. Arch Suicide Res.

[16] Nunavut Bureau of Statistics Nunavut suicides by region, sex, age group and ethnicity, 1999 to 2012 [cited 2014 Dec 29]. http://stats.gov.nu.ca/en/Population%20deaths.aspx.

[17] Wexler L, Hill R, Bertone-Johnson E, Fenaughty A (2008). Correlates of Alaska native fatal and nonfatal suicidal behaviours, 1990–2001. Suicide Life Threat Behav.

[18] Kirmayer LJ, Malus M, Boothroyd LJ (1996). Suicide attempts among Inuit youth: a community survey of prevalence and risk factors. Acta Pyschiatr Scand.

[19] Bjerregaard P, Curtis T (2002). Cultural change and mental health in Greenland. The association of childhood conditions, language and urbanization with mental health and suicidal thoughts among the Inuit of Greenland. Soc Sci Med.

[20] Eliassen BM, Melhus M, Kruse J, Poppel B, Broderstad AR (2012). Design and methods in a survey of living conditions in the Arctic – the SLiCA study. Int J Circumpolar Health.

[21] Saudny H, Leggee D, Egeland G (2012). Design and methods of the Adult Inuit Health Survey 2007–2008. Int J Circumpolar Health.

[22] Dene Nation (2012). First nations regional health survey report 2008–2010.

